# Heart Failure Admission Service Triage (H-FAST) Study: Racialized Differences in Perceived Patient Self-Advocacy as a Driver of Admission Inequities

**DOI:** 10.7759/cureus.13381

**Published:** 2021-02-16

**Authors:** Emily C Cleveland Manchanda, Regan H Marsh, Chidinma Osuagwu, Jennifer Decopain Michel, Julianne N Dugas, Michael Wilson, Michelle Morse, Eldrin Lewis, Bram P Wispelwey

**Affiliations:** 1 Department of Emergency Medicine, Boston Medical Center, Boston, USA; 2 Department of Emergency Medicine, Boston University School of Medicine, Boston, USA; 3 Department of Emergency Medicine, Brigham and Women’s Hospital, Harvard Medical School, Boston, USA; 4 Department of Medicine, Brigham and Women’s Hospital, Harvard Medical School, Boston, USA; 5 Division of Cardiology, Stanford University Medical Center, Palo Alto, USA

**Keywords:** heart failure, racial equity, emergency medicine, patient self-advocacy

## Abstract

Background

Racial inequities in mortality and readmission for heart failure (HF) are well documented. Inequitable access to specialized cardiology care during admissions may contribute to inequity, and the drivers of this inequity are poorly understood.

Methodology

This prospective observational study explored proposed drivers of racial inequities in cardiology admissions among Black, Latinx, and white adults presenting to the emergency department (ED) with symptoms of HF. Surveys of ED providers examined perceptions of patient self-advocacy, outreach to other clinicians (e.g., outpatient cardiologist), diagnostic uncertainty, and other active co-morbid conditions. Service census, bed availability, prior admission service, and other structural factors were explored through the electronic medical record.

Results

Complete data were available for 61/135 patients admitted with HF during the study period, which halted early due to coronavirus disease 2019. No significant differences emerged in admission to cardiology versus medicine based on age, sex, insurance status, education level, or perceived race/ethnicity. White patients were perceived as advocating for admission to cardiology more frequently (18.9 vs. 5.6%) and more strenuously than Black patients (p = 0.097). ED clinicians more often reported having spoken with the patient’s outpatient cardiologist for whites than for Black or Latinx patients (24.3 vs. 16.7%, p = 0.069).

Conclusions

Theorized drivers of racial inequities in admission service did not reach statistical significance, possibly due to underpowering, the Hawthorne effect, or clinician behavior change based on knowledge of previously identified inequities. The observed trend towards racial differences in coordination of care between ED and outpatient providers, as well as in either actual or perceived self-advocacy by patients, may be as-yet undemonstrated components of structural racism driving HF care inequities.

## Introduction

Racial inequities in mortality and readmission rates for patients with heart failure (HF) have been well documented in the literature [1-3]. Studies utilizing the Public Health Critical Race praxis operationalize Critical Race Theory to elucidate mechanisms through which structural racism creates these inequities and to build action plans accordingly [4-6]. At our large, urban, academic medical center, racial inequities in the admission of HF patients were identified retrospectively, demonstrating that Black and Latinx patients were disproportionately admitted to a general medicine service (GMS) rather than cardiology; the strongest predictor of admission to cardiology was having an established relationship with an in-network cardiologist [7]. Subsequently, a multidisciplinary working group hypothesized nine potential drivers of this manifestation of structural racism, including admission guidelines; hospital bed availability; cardiology service census; prior service to which the patient was admitted; having an in-network cardiologist; patient self-advocacy; outpatient provider advocacy; clinical uncertainty, including other active co-morbid conditions as well as complex social needs, concomitant psychiatric and/or substance use disorders at the time of admission; and clinician implicit bias. This study was designed to explore the impact of seven of these nine drivers, excluding admissions guidelines and implicit bias among providers, which are being examined in other work.

## Materials and methods

Patients presenting to the emergency department (ED) with symptoms suggestive of acute or acute-on-chronic HF who were subsequently admitted to either cardiology or GMS were prospectively identified at the end of each day through the electronic medical record (EMR) based on the chief complaint (e.g., shortness of breath, volume overload), diuretic medication administration in the ED, and admission diagnosis between October 7, 2019 and March 20, 2020. Patients who were subsequently found to have a clear alternative diagnosis for their symptoms (e.g., pneumonia) were excluded. An anonymous electronic survey was sent to the ED physician who admitted the patient to determine their degree of clinical certainty regarding the patient’s diagnosis, the patient’s perceived race and ethnicity, the perceived extent to which a patient or their family advocated for admission to a particular service, whether this self-advocacy influenced the clinician’s choice of admission service, whether the patient’s outpatient cardiologist was contacted prior to admission, and other factors theorized as contributors to the admission service decision. Clinicians were given up to 48 hours to complete the survey and, if needed, were reminded twice to do so within the timeframe. Additional patient and institutional data were captured through the EMR, including prior admission service for HF exacerbations, prior care from a cardiologist within our healthcare system, as well as hospital and cardiology service census at the time of admission. Descriptive statistics were used to summarize findings. Chi-square or Fisher’s exact tests were used to compare binary and categorical variables. Wilcoxon rank-sum tests were used for ordinal comparisons and non-parametric tests for non-normal continuous outcomes. Analysis was completed in SAS v9.4 (SAS Institute, Inc., Cary, NC, USA). This study was reviewed and approved by the local institutional review board (protocol 2019P001606).

## Results

A total of 135 patients with symptoms consistent with HF were admitted through the ED during the study period. Complete survey data from ED providers was available for 67 patients (49.6% response rate) within 48 hours of the clinical encounter. Data analysis was restricted to comparisons between patients perceived by clinicians as Black (n = 18), Latinx (n = 6), or white (n = 37), yielding a total study population of 61 patients. Five patients had missing race/ethnicity data; one was marked “other.” The racial and ethnic distribution of the study cohort is similar to that of the overall ED patient population cared for at our institution. The study did not reach the planned enrollment of 117 patients, as it was halted in mid-March 2020 when the coronavirus disease 2019 (COVID-19) pandemic dramatically altered ED patient volume and admission practices for patients presenting with shortness of breath and related symptoms.

Overall, 41 (67.2%) patients were admitted to cardiology while 20 (32.8%) were admitted to GMS. There were no significant differences in age (p = 0.091), sex (p = 0.772), insurance status (p = 0.705), education level (p = 0.373), or the patient’s perceived race/ethnicity (p = 0.942) between these groups (Table 1). For both white and non-white patients, HF was reportedly the dominant reason for admission; other active co-morbidities contributed to a lesser extent (Table 2). There was no significant difference by patient race/ethnicity in perceived unmet social needs (p = 0.636), psychiatric co-morbidities (p > 0.999), or substance use disorders (p = 0.200).

**Table 1:** Demographic comparison of patients presenting to the ED with symptoms of HF, by admission service. ED, emergency department; HF, heart failure; IQR, interquartile range

	Overall (n = 61)	Cardiology (n = 41)	Medicine (n = 20)	P-Value
Age, median (IQR)	74.0 (21.0)	77.0 (18.0)	67.0 (23.5)	0.0908
Sex, n (%)				0.7723
Female	35 (57.4)	23 (56.1)	12 (60.0)	
Male	26 (42.6)	18 (43.9)	8 (40.0)	
Patient’s perceived race/ethnicity, n (%)				0.9416
Black or Hispanic/Latinx	24 (39.3)	16 (39.0)	8 (40.0)	
White	37 (60.7)	25 (61.0)	12 (60.0)	
Insurance, n (%)				0.7048
Commercial	22 (36.1)	14 (34.1)	8 (40.0)	
Government	38 (62.3)	26 (63.4)	12 (60.0)	
Missing	1 (1.6)	1 (2.4)	0 (0.0)	
Education level, n (%)				0.3727
8th grade or less	3 (4.9)	2 (4.9)	1 (5.0)	
Some high school	4 (6.6)	3 (7.3)	1 (5.0)	
High school graduate or GED	19 (31.1)	9 (22.0)	10 (50.0)	
Some college or 2-year degree	7 (11.5)	5 (12.2)	2 (10.0)	
4-year college degree	18 (29.5)	13 (31.7)	5 (25.0)	
More than 4-year college degree	5 (8.2)	5 (12.2)	0 (0.0)	
Other	1 (1.6)	1 (2.4)	0 (0.0)	

White patients were perceived as advocating for admission to a given service more frequently (18.9 vs. 5.6%) and more strenuously than Black patients, which trended toward but did not reach statistical significance (p = 0.097, data not shown). However, this trend did not persist when comparing white to non-white patients (p = 0.278, see Table 2). Among patients who expressed a preference, physicians reported that patient self-advocacy contributed to their choice of admission service for six of the seven white patients (85.7%) compared to only one (50%) non-white patient (p = 0.440). There was no significant racial difference in having an in-network cardiologist (p = 0.17). However, among the 43 patients with an in-network cardiologist, clinicians were more likely to report having spoken with this outpatient provider for white patients than for Black or Latinx patients (24.3 vs. 16.7%, p = 0.069).

**Table 2:** Comparison of proposed drivers of admission decisions, by race/ethnicity. CHF, congestive heart failure; ED, emergency department *for Black versus white patients, p = 0.0969

	Overall (n = 61)	Black/Latinx (n = 24)	White (n = 37)	P-Value
To what extent was heart failure the reason for admission?, n (%)				0.448
Not at all	5 (9.1)	2 (11.1)	3 (8.1)	
To a small extent	3 (5.5)	1 (5.6)	2 (5.4)	
To some or a moderate extent	10 (18.2)	3 (16.7)	7 (18.9)	
To a great or very great extent	37 (67.3)	12 (66.7)	25 (67.6)	
To what extent were the patient’s other (non-CHF) active co-morbidities the reason for the patient’s admission?, n (%)				0.575
Not at all	7 (11.5)	4 (16.7)	3 (8.1)	
To a small extent	3 (4.9)	1 (4.2)	2 (5.4)	
To some or a moderate extent	11 (18.0)	4 (16.7)	7 (18.9)	
To a great or very great extent	40 (65.6)	15 (62.5)	25 (67.6)	
Did the patient have significant apparent unmet social needs (e.g., homelessness)?, n (%)				0.636
Yes	1 (1.6)	1 (4.2)	0 (0.0)	
No	59 (96.7)	23 (95.8)	36 (97.3)	
Unknown	1 (1.6)	0 (0.0)	1 (2.7)	
Did the patient have significant psychiatric co-morbidities?, n (%)				>0.999
Yes	0 (0.0)	0 (0.0)	0 (0.0)	
No	58 (95.1)	22 (91.7)	36 (97.3)	
Unknown	2 (3.3)	1 (4.2)	1 (2.7)	
Missing	1 (1.6)	1 (4.2)	0 (0.0)	
Did the patient have significant apparent substance use?, n (%)				0.200
Yes	6 (9.8)	4 (16.7)	2 (5.4)	
No	55 (90.2)	20 (83.3)	35 (94.6)	
Did the patient and/or their family indicate a preference for admission location?, n (%)				0.278*
Not at all	47 (77.0)	19 (79.2)	28 (75.7)	
To a small extent	3 (4.9)	1 (4.2)	2 (5.4)	
To some or a moderate extent	3 (4.9)	1 (4.2)	2 (5.4)	
To a great or very great extent	3 (4.9)	0 (0.0)	3 (8.1)	
Missing	5 (8.2)	3 (12.5)	2 (5.4)	
If any preference was indicated, to what extent did the patient/family preference weigh into your decision for location of admission?, n (%)				0.440
Not at all	2 (22.2)	1 (50.0)	1 (14.3)	
To a small extent	1 (11.1)	0 (0.0)	1 (14.3)	
To some or a moderate extent	4 (44.4)	1 (50.0)	3 (42.9)	
To a great or very great extent	2 (22.2)	0 (0.0)	2 (28.6)	
What was the cardiology census was at the time of admission?, n (%)				0.439
Regular	24 (39.3)	8 (33.3)	16 (43.2)	
Extreme	37 (60.7)	16 (66.7)	21 (56.8)	
Does the patient have a Partner Cardiologist?, n (%)				0.1170
Yes	37 (60.7)	12 (50.0)	25 (67.6)	
No	24 (39.3)	12 (50.0)	12 (32.4)	
Did you speak to the patient’s cardiologist regarding the patient’s presentation to the ED or the decision to admit?, n (%)				0.069
Yes	13 (21.3)	4 (16.7)	9 (24.3)	
No	30 (49.2)	13 (54.2)	17 (45.9)	
Not applicable/missing	18 (29.5)	7 (29.2)	11 (29.7)	

## Discussion

This prospective study implemented the Public Health Critical Race praxis to explore the proposed drivers of previously identified racial inequities in access to specialized cardiology care for patients presenting to the ED with symptoms of HF. Of the theorized drivers, none were found to be significantly different between Black or Latinx and white patients. This may be due to underpowering as the study was halted when the COVID-19 pandemic affected ED patient volumes and shifted admission patterns. Alternatively, improved equity in admission patterns may reflect the Hawthorne effect as the results of the retrospective study highlighting the historical inequities were widely known prior to the study’s initiation.

Several interesting findings are worthy of mention. Despite similar proportions of having an in-network cardiologist, we observed a trend towards ED clinicians contacting outpatient cardiologists more frequently for white patients than for Black and Latinx patients. As the question regarding contact with an outpatient cardiologist was only asked if the patient had previously been identified as having an in-network cardiologist, it is unlikely that the ease of contacting this provider would differ significantly between patients. The increased frequency of contact with outpatient cardiologists of white patients may be secondary to individual level factors, for example, unconscious racial bias among ED clinicians making them more likely to engage outpatient providers to coordinate care for white patients. Structural factors may also play a role. Black and Latinx patients may have less frequent outpatient cardiology appointments [8], potentially due to adverse social determinants of health limiting healthcare system engagement [9]. ED clinicians may conclude that the outpatient provider is less likely to have relevant information to contribute to admission decisions. It is not possible from this study to determine whether or to what extent the greater frequency with which ED physicians contacted outpatient cardiologists for white patients influenced decision-making for these patients. However, engagement with outpatient providers prior to admission may increase the likelihood of cardiology admission and represents an important area for further study.

Second, there was a trend towards clinicians perceiving that white patients advocated for admission to the cardiology service more frequently and strenuously than Black patients. This perception may have influenced admission decisions, and would signal a previously unrevealed mechanism for racial inequities in the healthcare setting. Prior studies have demonstrated that clinicians poorly predict patient treatment preferences [10], which may be influenced by race, gender, education level, and other factors [11]. Some data support that eliciting patient preference may positively influence care, including outcomes [12] and satisfaction [13]. However, few studies have explored patient preference for admission service, and we are not aware of any studies exploring racial differences in this facet of patient agency.

Our findings raise important questions about racialized differences in both actual and perceived patient agency. White patients may be able to more effectively communicate a stronger preference for the type of care they receive, which may in turn lead to increased access to specialized care, and thus, represents an example of operationalized white privilege in the healthcare system. Greater or more effective self-advocacy by white patients may stem from increased agency, knowledge, and ease in navigating a complex medical system built towards the needs of white patients. Additionally, ED physicians, who are predominantly white [14], may be more comfortable eliciting or sensitized to perceiving preferences from white patients than patients of color [15].

## Conclusions

This study revealed a trend toward racial differences in coordination of care between ED and outpatient providers, as well as in either actual or perceived self-advocacy by patients, which may be as-yet undemonstrated components of structural racism driving HF care inequities. Our findings suggest the need to further explore factors that shape clinician and patient preference for admission service, provider perceptions of patient self-advocacy, and the impact that these have on clinical decision-making and outcomes.
